# Ligand Based Pharmacophore Modeling and Virtual Screening Studies to Design Novel HDAC2 Inhibitors

**DOI:** 10.1155/2014/812148

**Published:** 2014-11-26

**Authors:** Naresh Kandakatla, Geetha Ramakrishnan

**Affiliations:** Department of Chemistry, Sathyabama University, Jeppiaar Nagar, Chennai 600119, India

## Abstract

Histone deacetylases 2 (HDAC2), Class I histone deacetylase (HDAC) family, emerged as an important therapeutic target for the treatment of various cancers. A total of 48 inhibitors of two different chemotypes were used to generate pharmacophore model using 3D QSAR pharmacophore generation (HypoGen algorithm) module in Discovery Studio. The best HypoGen model consists of four pharmacophore features namely, one hydrogen bond acceptor (HBA), and one hydrogen donor (HBD), one hydrophobic (HYP) and one aromatic centres, (RA). This model was validated against 20 test set compounds and this model was utilized as a 3D query for virtual screening to validate against NCI and Maybridge database and the hits further screened by Lipinski's rule of 5, and a total of 382 hit compounds from NCI and 243 hit compounds from Maybridge were found and were subjected to molecular docking in the active site of HDAC2 (PDB: 3MAX). Finally eight hit compounds, NSC108392, NSC127064, NSC110782, and NSC748337 from NCI database and MFCD01935795, MFCD00830779, MFCD00661790, and MFCD00124221 from Maybridge database, were considered as novel potential HDAC2 inhibitors.

## 1. Introduction

Histone deacetylases (HDACs) are the enzymes that deacetylase the epsilon-N-acetyl-lysine group on histone tails of the protein and result in tightening of nucleosome structure and gene silencing [1]. There are two types of histone forms which are histone acetylases and histone deacetylases [2]. Histone deacetylases (HDACs) are found in animals, plants, fungi, archaebacteria, and eubacteria [3]. Histone deacetylases are generally classified into four different classes, namely, HDACs 1–3 and 8, belonging to Class I and related to homologous to Rpd3, HDAC 4–7, 9-10 are Class II related to Hda1, Sirt 1–7 are Class III and are similar to Sir2 and HDAC11 belongs to Class IV. Classes I and II are operated by zinc dependent mechanism and Class III by NAD [4–8]. Histone deacetylases (HDACs) control the gene expression and cellular signaling and histone deacetylases 2 (HDAC2) is overexpressed in solid tumors including colon cancer, lung cancer, cervical carcinoma, breast cancer, and kidney/cervix cancer and also in Alzheimer's disease [9, 10]. Several HDAC inhibitors are in clinical trial, namely, hydroxamic acid derivatives, benzamide derivatives, cyclic peptides, and short-chain fatty acids [11]. The first histone deacetylase (HDAC) inhibitor SAHA (suberoylanilide hydroxamic acid or vorinostat) approved by FDA for treating cutaneous T-cell lymphoma and other hydroxamic acids are in clinical trial. The benzamide derivatives, which are in clinical trials, are Entinostat (MS-275 or pyridin-3-yl methyl 4-((2-aminophenyl) carbamoyl) benzyl carbamate) currently in phase II clinical trial for Hodgkin lymphoma, phase I trial of advanced leukemia and myelodysplastic syndrome (MDS), and Mocetinostat (MGCD0103 or N-(2-Aminophenyl)-4-[[(4-pyridin-3-ylpyrimidin-2-yl)amino]methyl] benzamide) in phase II clinical trial for Hodgkin lymphoma, phase I trial of advanced leukemia, myelodysplastic syndrome (MDS), diffuse large B-cell lymphoma, and follicular lymphoma [12–15]. Ligand based pharmacophore modeling is a major tool in drug discovery and is applied in virtual screening, de novo design, and lead optimization [16]. Different histone deacetylase (HDAC) inhibitors had been synthesized and experimental activity was found. Different pharmacophore and virtual screening studies had been reported on histone deacetylase (HDAC) with known hydroxamic acid derivatives and QSAR studies reported on histone deacetylases 2 (HDAC2) with N(2-aminophenyl)-benzamides [17–19]. In the present study benzamide derivatives are used to generate the pharmacophore model and virtual screening studies have been done for histone deacetylases 2 (HDAC2) proteins to gain knowledge regarding pharmacophore model and virtual screening. This study aims to construct the chemical feature based on pharmacophore models for histone deacetylases 2 (HDAC2).

## 2. Materials and Methods

### 2.1. Data Preparation

A training set of 48 histone deacetylases 2 (HDAC2) inhibitors of two different chemotypes were selected form previously published data and the IC_50_ values were identified using the same biological assay. The chemotype A is N(2-aminophenyl)-benzamide [20–31] and chemotype B is N-hydroxy benzamide derivatives (see supplementary Figure 1 in the Supplementary Material available online at http://dx.doi.org/10.1155/2014/812148) [32–34]. 3D QSAR module in Discovery Studio (DS) was used for developing the pharmacophore. The 2D structure of compounds was drawn in ISIS draw and they were converted into 3D form and conformational models were generated by FAST method, the conformers minimized by the CHARMm force field and the energy threshold value of 20 kcal/mol. A maximum of 255 conformers were developed for each compound and these conformer models were used for hypotheses generation, fitting the compound into the hypotheses and estimating the activity of the compound. The training set of 48 molecules was chosen with IC_50_ values with a range from 0.014 *μ*M to 21 *μ*M. The dataset activity (IC_50_) was classified based on the span over four orders of magnitude, that is, active (IC_50_ ≤ 0.1 *μ*M, ++++), moderately active (0.1 ≤ IC_50_ ≤ 1 *μ*M, +++), less active (1 ≤ IC_50_ ≤ 10 *μ*M, ++), and inactive (IC_50_ > 10 *μ*M, +).

### 2.2. Pharmacophore Model Generation

HypoGen algorithm was applied to build the pharmacophore model and in the present study four features, which are hydrogen bond donors (HBD), hydrogen bond acceptors (HBA), ring aromatic (RA), and hydrophobic (HY), were selected to generate the pharmacophore hypotheses [35]. HypoGen generates pharmacophore model based on chemical features of active compounds in training set. The uncertainty value 2 was selected from default 3, which means the biological activity is two times higher or lower than the true value. All other parameters were kept as default. The developed pharmacophore model was selected based on the highest correlation coefficient, lowest total cost, and root mean square deviation (RMSD).

### 2.3. Pharmacophore Validation

The pharmacophore model is validated by three steps: cost analysis, Fischer's randomization test, and the test set prediction. The quality of the model is described in terms of fixed cost, total cost, and null cost. The fixed cost represents the simplest model and it fits the data perfectly. The null cost represents no features with high cost value and it estimates the activity to be average activity of the training set compounds. The best model was selected based on the difference between the two cost values (null cost − total cost); if the difference between the costs is greater than 60 means, the model has excellent true correlation. If the difference is 40–60, the model has prediction correlation of 70–90%, and if the difference is below 40, it may be difficult to predict the model. Fischer's randomization is the second approach to validate the pharmacophore model. The 95% confidence level was selected to validate the study and 19 random spread sheets were constructed. This method generates the hypotheses by randomizing the activity of the training set compounds. The correlation between the structure and biological activity was validated by this method. The final sets of validation were selected using twenty HDAC2 inhibitors as given in supplementary Figure 2. The Ligand pharmacophore mapping module in Discovery Studio was used to map the ligands and estimate the predicted activity of the test set compounds.

### 2.4. Database Search

Virtual screening studies were used to find novel and potential leads from virtual database for further development [36]. The virtual screening studies were used to find novel leads for HDAC2. The Hypo1 model was used as a 3D query in database screening, and the National Cancer Institute (NCI) database containing 265242 molecules and Maybridge database containing 58723 molecules were used for screening [37, 38]. Ligand pharmacophore mapping protocol was used with flexible search option to screen the database. Hit compounds from the database with estimated activity less than 0.1 *μ*M were selected for further screening using Lipinski's rule of five; compounds have (i) molecular weight less than 500, (ii) hydrogen donors less than 5, (iii) hydrogen acceptors less than 10, and (iv) an octanol/water partition coefficient (Log*P*) value less than 5.

### 2.5. Molecular Docking

Docking is the binding orientation of small molecules to their protein targets in order to predict the affinity and activity of the small molecules. Hence docking plays an important role in the rational drug design. Molecular docking studies were performed by using LigandFit module in Discovery Studio [39]. There are three stages in LigandFit protocol: (i) docking, in which attempt is made to dock a ligand into a user defined binding site, (ii) in situ ligand minimization, and (iii) scoring, in which various scoring functions were calculated for each pose of the ligands. Protein preparation was the main step in docking and all ligands were docked into the active site of the receptor. Protein preparation involves deletion of water molecules and addition of hydrogen atoms and applying CHARMm force field. The active sites were searched using flood filling algorithm. The active site was defined as region of HDAC2 that comes within 12 Å from the geometric centroid of the ligand. Ten poses were generated for each ligand during the docking process and the best poses were selected based on the best orientation of the molecule in the active site and dock score values, which was selected after energy minimization with smart minimization. The dock score was calculated using the following formula:

(1) DockScore  (force  field)=−ligandreceptor  interaction  energy+ligand  internal  energy.

Single dock score may fail to obtain active molecules; hence, consensus scoring method was applied which consists of LigScore1, LigScore2, Jain, Piecewise Linear Potential (PLP1 and PLP2), and Potential of Mean Force (PMF). The active molecules were selected based on the consensus scoring method and H-bond interaction with the receptor. The crystal structure of the HDAC2 protein (PDB ID: 3MAX) was downloaded from the protein data bank (http://www.rcsb.org/pdb). The crystal structure of histone deacetylases 2 (HDAC2) protein has three chains, which are A, B, and C. The chain A has higher docking score than chains B and C, so chain A is selected for docking. The hit compounds from the database screening with positive Lipinski's drug likeness were subjected to molecular docking studies into the active site of the 3MAX receptor.

## 3. Results and Discussion

### 3.1. Pharmacophore Generation

**Figure 1:**
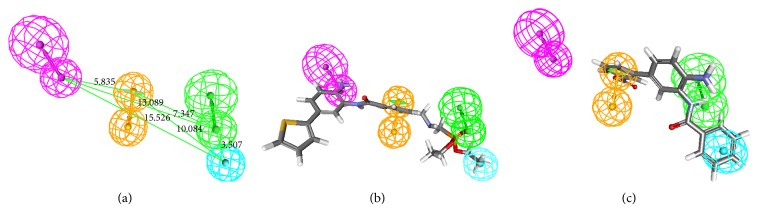
The best pharmacophore model (Hypo1) of HDAC2 inhibitors generated by the HypoGen module: (a) the best pharmacophore model Hypo1 represented with distance constraints (Å), (b) Hypo1 mapping with one of the active compounds 15, and (c) Hypo1 mapping with one of the least active compounds 29. Pharmacophoric features are colored as follows: hydrogen bond acceptor (green), hydrogen bond donor (magenta), hydrophobic (cyan), and ring aromatic (orange).

Pharmacophore model, virtual screening, and molecular docking studies were performed to find novel HDAC2 inhibitors. Dataset of 48 molecules with structural diversity and four orders of activity magnitude (0.014 to 21 *μ*M) were selected to develop pharmacophore model using HypoGen algorithm in Discovery Studio. Four features hydrogen bond donor (HBD), hydrogen bond acceptors (HBA), ring aromatics (RA), and hydrophobic (HY) were selected. Top 10 hypotheses were generated with the following features: HBA, HBD, RA, and HY. The statistical parameters such as cost values, correlation, and RMSD were summarized in Table 1. The best hypothesis was selected out of 10 hypotheses by the highest cost difference. Hypo1 has the highest cost difference between null cost and total cost of 68.45, correlation coefficient of 0.75, the lowest RMS deviation of 1.64, and configuration cost value of 17.66. This indicates the model and the data correlated by more than 90%. High correlation coefficient and low RMSD indicate the ability to predict the activity of the training set compounds is high. The Hypo1 has a correlation coefficient value (*R* ^2^ = 0.75), and the model strongly predicts the activity of training set compounds. The correlation between the experimental activity and predicted activity of training set compounds was shown in Table 2. For most of the compounds the model predicts the activity correctly.  Figure 1(a) shows the 3D spatial arrangement of all features with distance constraints of Hypo1. The features of Hypo1 were mapped onto the active compound 15 as shown in Figure 1(b). HBD is mapped by amino group, HBA is mapped by phosphonate group, aromatic ring is mapped by aromatic group, and HY is mapped by hydrophobic group. The results indicate that HDAC2 inhibition requires the following features: HBD, HBA, RA, and HYP. Inactive compound 29 was mapped partially onto the features of Hypo1 as shown in Figure 1(c). The fit value for the most active and the least active compounds was generated to be 5.39 and 3.21, respectively.

### 3.2. Pharmacophore Validation

The pharmacophore model can be validated by three methods: cost analysis, test set prediction, and Fischer's randomization test.

#### 3.2.1. Cost Analysis

The HypoGen algorithm in DS produces three cost values during the pharmacophore generation, which are fixed cost, total cost, and null cost. The model is validated by the difference between the null cost and total cost; if the model has cost difference above 60, it has the predictability chance of greater than 90%. The Hypo1 having the cost difference of 68.45 shows significant model (shown in Table 1).

#### 3.2.2. Test Set Prediction

A good pharmacophore model can predict not only the activity of the training set compounds but also external test set compounds. 20 compounds with different activity range were used as a test set to check the predictability power of the pharmacophore model. Ligand pharmacophore mapping protocol with flexible search option was used to map the test set compounds. In test set analysis, for most of the compounds the model predicted activity to the tune of less than 10%. Out of 20 compounds 17 compounds were predicted with an error factor less than 5% and 3 compounds were predicted with an error factor less than 10%. The experimental and predicted activities of the test set compounds were shown in Table 3.

#### 3.2.3. Fischer Randomization Test

**Figure 2:**
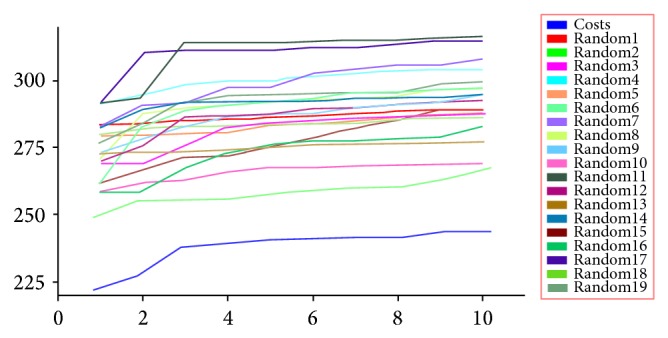
Fischer randomization test for 95% confidence level: pharmacophore hypotheses versus total cost.

Fischer randomization test was the third approach to validate the Hypo1 using DS. In this method the experimental activity of the training set compounds was randomly scrambled and generates the random pharmacophore model using the same parameters as used in developing the Hypo1 hypothesis. Confidence level of 95% was set and it created 19 spread sheets, all 19 random spread sheets have high cost values than total cost, and correlation value is less than the Hypo1 (supplementary Table 1). It clearly shows none of the randomly generated pharmacophores has good statistical values than Hypo1. The difference in costs between the HypoGen and Fischer randomizations was shown in Figure 2. All the three validations methods demonstrated that Hypo1 hypothesis has good predictability and can be chosen as the best model.

### 3.3. Database Screening

**Figure 3:**
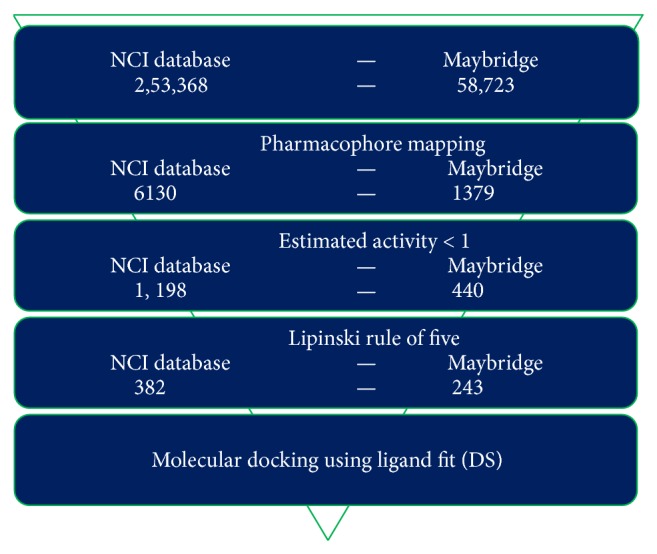
Schematic representation of virtual screening process implemented in the identification of HDAC2 inhibitors.

The best pharmacophore model Hypo1 was used as a 3D query to search the NCI (265242) and Maybridge (58723) databases using flexible search option in DS. A total of 6130 compounds from NCI and 1379 from Maybridge were mapped using the features of Hypo1. A total of 1198 and 440 compounds from NCI and Maybridge showed HypoGen estimated value of less than 1 *μ*M and were considered for further studies and these compounds were screened for Lipinski's rule of 5. A total of 625 (382 NCI, 243 Maybridge) compounds obeyed the rule and were subjected to molecular docking studies. The flowchart in Figure 3 was a schematic representation of virtual screening process.

### 3.4. Molecular Docking

**Figure 4:**
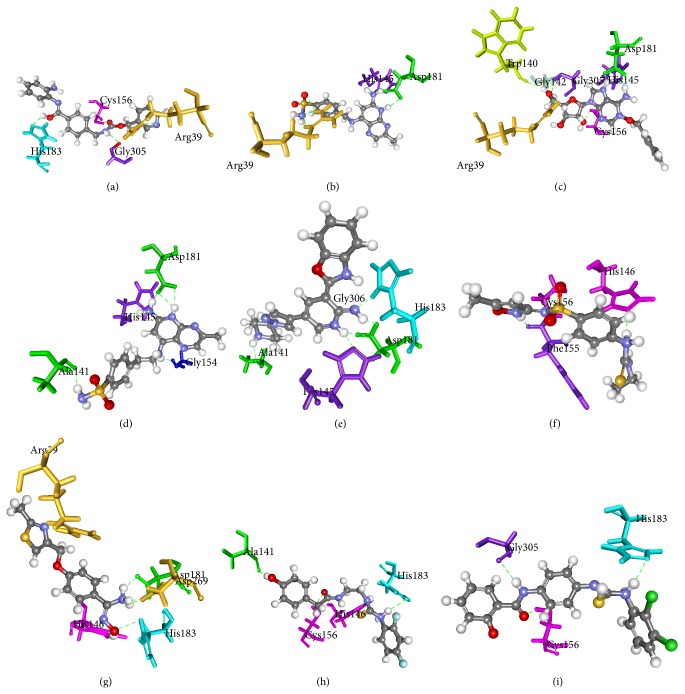
Binding orientations of hit compounds: (a) Entinostat, (b) NSC108392, (c) NSC127064, (d) NSC110782, (e) NSC748337, (f) MFCD01935795, (g) MFCD00830779, (h) MFCD00661790, and (i) MFCD00124221 in the active site of 3MAX-A with hydrogen bond interactions.

The HDAC2 protein has three chains which are A, B, and C. The active compound MS-275 (Entinostat) was docked into active sites of all three chains using LigandFit module in Discovery Studio, and out of three chains chain A has given the best docking score and higher H-bond interactions than chains B and C. The docking score of all three chains with Entinostat was shown in supplementary Table 2. Chain A was selected as an active chain and the final hit compounds from virtual screening studies were docked into active site of 3MAX-A. The docking score along with binding orientations and hydrogen bonds was considered for choosing the best pose of the docked compounds. The docking scores were compared with MS-275 (Entinostat). The docking score of the Entinostat was 42.6 kcal/mol and hit compounds from the virtual screening studies show better binding than the active compound Entinostat. The Entinostat has the four hydrogen bonding interactions with Arg39, Cys156, Gly305, and His183 given in Figure 4(a). The hit compounds that scored docking score higher than active compound and form interaction with the crucial amino acids were considered as effective leads for designing novel HDAC2 inhibitors. 74 compounds from both databases showed good interactions in the active site of the HDAC2 and scored more than 45, about 20 compounds that showed better docking score than active compounds were chosen as leads and their docking score and H-bond interactions were listed in supplementary Tables 3 and 4. Finally four compounds from NCI, namely, NSC108392, NSC127064, NSC110782, and NSC748337, were identified with good docking score and estimated activity value of 0.26 *μ*M, 0.47 *μ*M, 0.37 *μ*M, and 0.41 *μ*M, respectively. The hit NSC108392 (4-(((6-amino-3-methylpyrido[2,3-b]pyrazin-8-yl)amino)methyl)benzenesulfonamide) has the docking score of 121.9 kcal/mol and forms three hydrogen bond interactions with Arg39 (3), His145, and Asp181 (2) amino acids shown in Figure 4(b). The binding mode of this compound at the active site showed that mapping on HBD feature of Hypo1 formed interactions with Arg39 and HBA feature of Hypo1 forms the interactions with His145 and Asp181. NSC127064 ((2S,3S,4S,5R)-2-(1-(benzyloxy)-6-imino-1H-purin-9(6H)-yl)-5-(hydroxymethyl) tetrahydrofuran-3,4-diol) has the docking score of 116.4 kcal/mol and forms seven hydrogen bond interactions with Arg39, Cys156, Gly305, His145 (2), Asp181 (2), Trp140, and Gly142 amino acids shown in Figure 4(c). The binding mode and pharmacophore overlay of the compound showed that OH mapped on HBD, NH2 mapped on HBA, and purine moiety mapped on RA form interaction in the active site. NSC110782 (4-(2-((6-amino-3-methylpyrido[2,3-b]pyrazin-8-yl) amino) ethyl) benzene sulfonamide) has the docking score of 106.2 kcal/mol and forms four hydrogen bond interactions with His145, Asp181 (3), Gly154, and Ala141 shown in Figure 4(d). The binding mode and pharmacophore overlay of this compound showed that HBD mapped on NH2 and HBA mapped on pyrazine moiety have interactions with the amino acid residues. NSC748337 (3-(benzo[d]oxazol-2-yl)-5-(1-(piperidin-4-yl)-1H-pyrazol-4-yl) pyridin-2-amine) has the docking score of 105.6 kcal/mol and forms four hydrogen bond interactions with Asp181 (2), His145, Ala141, and His183 shown in Figure 4(e). The nitrogen on piperidine mapped to HBD has interactions with Ala141, amino group on pyridine moiety mapped to HBA of Hypo1 has interactions with His183, and Asp181 and nitrogen of pyridine moiety have interactions with His145. Four compounds from Maybridge database, MFCD01935795, MFCD00830779, MFCD00661790, and MFCD00124221, were identified as novel HDAC2 inhibitors with estimated activity value of 0.12 *μ*M, 0.32 *μ*M, 0.61 *μ*M, and 0.68 *μ*M, respectively. MFCD01935795 (4-(3-ethylthioureido)-N-(5-methylisoxazol-3-yl) benzenesulfonamide) as the docking score of 98.8 kcal/mol having three H-bond interactions with Cys156, Phe155, and His146 residues is shown in Figure 4(f). The protein-ligand interaction shows HBD of Hypo1 forms hydrogen bond interaction with His146, and nitrogen on iso-oxazole mapped with HBA forms hydrogen bond with Phe155 and oxygen of sulphonamide forms bond with Cys156. MFCD00830779 ((E)-N′-hydroxy-4-((2-methylthiazol-4-yl)methoxy)benzimidamide) having the docking score of 96.89 kcal/mol forms five hydrogen bonds with Arg39, His183, Asp181 (2), Asp269, and His146 amino acids. The binding mode of the compound shown in Figure 4(g) shows Hypo1 of HBA mapped nitrogen of thiazole forms hydrogen bonding with Arg39, HBD mapped on oxygen of N-hydroxy has hydrogen bond interaction with His183, benzamide of nitrogen has interaction with His146, and nitrogen of formamide has interaction with Asp181. MFCD00661790 (N-(2-(3-(2,4-difluorophenyl)thioureido)ethyl)-2-(4-hydroxyphenyl)acetamide) has the docking score of 81. Kcal/mol forming four hydrogen bond interactions with Cys156, His183, His146, and Ala141 amino acids, Hypo1 HBD mapped on oxygen shows hydrogen bond interaction with Ala141, HBA mapped on nitrogen forms bonds with His183, nitrogen of thiourea forms bonds with His146, and acetamide of oxygen has hydrogen bond with Cys156; the binding interactions are shown in Figure 4(h). The fourth hit MFCD00124221 (N-(4-(3-(2,3-dichlorophenyl)thioureido)phenyl)-2-hydroxybenzamide) has docking score of 65.86 kcal/mol with three hydrogen bonds with Cys156, Gly305, and His183. The complex shown in Figure 4(i) shows benzamide of oxygen with Cys156, nitrogen with Gly305, and nitrogen of thiourea with His183 and shows hydrogen bond interaction.

**Figure 5:**
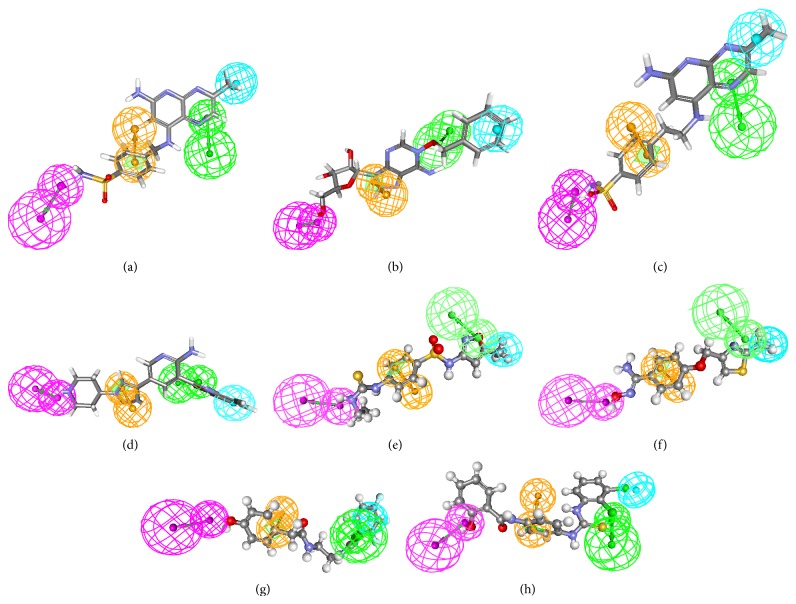
The pharmacophore overlay of hit compounds: (a) NSC108392, (b) NSC127064, (c) NSC110782, (d) NSC748337, (e) MFCD01935795, (f) MFCD00830779, (g) MFCD00661790, and (h) MFCD00124221.

**Figure 6:**
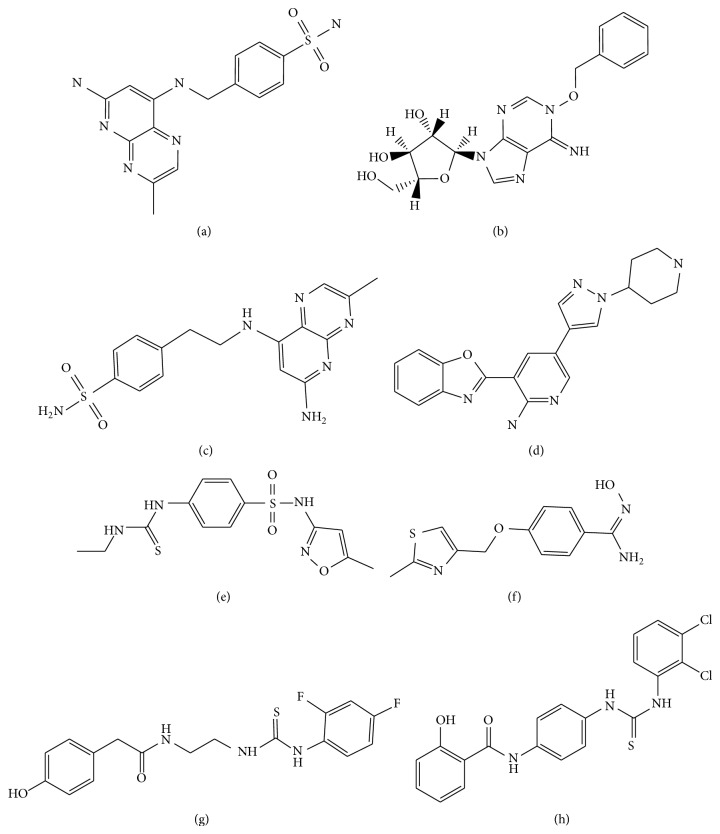
Identified lead compounds through NCI and Maybridge database search: (a) NSC108392, (b) NSC127064, (c) NSC110782, (d) NSC748337, (e) MFCD01935795, (f) MFCD00830779, (g) MFCD00661790, and (h) MFCD00124221.

The pharmacophore overlay of the hit compounds was shown in Figure 5. The identified lead compounds along with their estimated IC_50_ were shown in Figure 6. The studies show Arg39, Cys156, His145, and His146 were the important amino acids in the active site involved in hydrogen bond interaction. Based on pharmacophore modeling, virtual screening, and molecular docking studies, the compounds listed in supplementary Table 3 are selected as novel leads for effective HDAC2 inhibition. All identified hits were with diverse scaffolds and provide opportunities for designing novel HDAC2 inhibitors. The lead compounds were selected based on the docking score and structural diversity. The correlation between the estimated activity and docking score of top 10 lead compounds from NCI and Maybridge is 0.61 and 0.54, respectively, which suggests that the selected inhibitors in the present study could be specific HDAC2 inhibitors.

Comparative study on previous developed models with present study shows common pharmacophore features were present in all studies. Pharmacophore model on histone deacetylase (HDAC) with known hydroxamic acids and cyclic peptides shows four pharmacophore features: one hydrogen acceptor and one hydrophobic and two aromatic rings [17] and in the study with known hydroxamic acids, benzamides, and biphenyl derivatives on HDAC [40] three pharmacophore features were shown: hydrogen acceptors, hydrogen donors, and hydrophobic aromatic ring. Pharmacophore model on histone deacetylase (HDAC8) with known hydroxamic acids has shown four pharmacophore features: one hydrogen acceptor, two hydrogen donors, and one hydrophobic group [18]. The present developed pharmacophore model on HDAC2 with known benzamide derivatives shows four pharmacophore features: one hydrogen acceptor, one hydrogen donor, and one hydrophobic and one aromatic rings, which correlates with the previous studies.

The selected eight lead compounds and Entinostat were docked into the active sites of histone deacetylase (HDAC) (PDB: 1ZZ1) and histone deacetylase (HDAC8) (PDB: 1T64), and in both the receptors chain A was selected for docking. The docking result shows that compounds with HDAC and HDAC8 comparably showed lesser docking score and interactions than HDAC2. The docking scores and H-bond interactions were shown in supplementary Tables 5 and 6.

The combinations of pharmacophore, virtual screening, and molecular docking successfully give more potential inhibitors that can have great impact for future experimental studies in diseases associated with HDAC2 inhibition.

## 4. Conclusion

In this study, ligand pharmacophore model developed by HypoGen algorithm in DS, 10 hypotheses were generated using 48 training set compounds with structural diversity. The best pharmacophore Hypo1 was characterized by high cost difference and correlation coefficient comprised of HBD, HBA, RA, and HY features. The Hypo1 was validated by external test set and Fisher's randomization test suggests the model has good predictability. The Hypo1 was used as a 3D query to screen NCI and Maybridge databases. 625 compounds with estimated activity less than 1 *μ*M and favourable Lipinski's rule were selected for docking studies. In molecular docking studies the important interactions with inhibitors and active site residues were determined. Based on docking score and interactions twenty hits were found and finally eight hits NSC108392 (4-(((6-amino-3-methylpyrido[2,3-b]pyrazin-8-yl)amino)methyl)benzenesulfonamide), NSC127064 ((2S,3S,4S,5R)-2-(1-(benzyloxy)-6-imino-1H-purin-9(6H)-yl)-5-(hydroxymethyl)tetrahydrofuran-3,4-diol), NSC110782 (4-(2-((6-amino-3-methylpyrido[2,3-b]pyrazin-8-yl)amino)ethyl)benzenesulfonamide), NSC748337 (3-(benzo[d]oxazol-2-yl)-5-(1-(piperidin-4-yl)-1H-pyrazol-4-yl)pyridin-2-amine), MFCD01935795 (4-(3-ethylthioureido)-N-(5-methylisoxazol-3-yl) benzenesulfonamide), MFCD00830779 ((E)-N′-hydroxy-4-((2-methylthiazol-4-yl)methoxy)benzimidamide), MFCD00661790 (N-(2-(3-(2,4-difluorophenyl)thioureido)ethyl)-2-(4-hydroxyphenyl)acetamide), and MFCD00124221 (N-(4-(3-(2,3-dichlorophenyl)thioureido)phenyl)-2-hydroxybenzamide) were selected based on structural diversity and stability. These novel compounds can be used for experimental studies for the inhibition of HDAC2.

## Supplementary Material

Supplementary figures 1 and 2 provides the chemical structures of training and test set compounds, Supplementary Table 1 provide the randomization test results, Supplementary Table 2-4 provides the docking score, H-bond interactions and length, amino acid residues of Entinostat and 20 lead compounds on HDAC2 receptor and Supplementary Table 5-6 provides the docking score, H-bond interactions and length, amino acid residues of Entinostat and 8 lead compounds on HDAC and HDAC8 receptor.

## Figures and Tables

**Table 1:** Statistical results of the generated pharmacophore models.

Hypo	Total cost	Cost difference^a^	RMS	Error cost	Correlation	Max fit	Features
1	223.598	68.459	1.64	204.016	0.759	6.4	HBA HBD HYP RA
2	228.662	62.395	1.7	209.498	0.735	6.9	HBA HBD HYP RA
3	238.771	53.286	1.82	219.538	0.689	6.86	HBA HYP RA RA
4	240.0	52.057	1.84	220.97	0.682	7.16	HBA HYP RA RA
5	241.524	50.533	1.84	221.036	0.682	5.77	HBA HYP HYP RA
6	241.71	50.347	1.86	222.568	0.674	6.98	HBA HYP RA RA
7	242.261	49.796	1.86	222.428	0.675	6.25	HBA HYP RA RA
8	242.339	49.718	1.85	221.941	0.677	5.83	HBA HYP RA RA
9	244.381	47.676	1.89	225.13	0.662	6.84	HBA HYP RA RA
10	244.543	47.514	1.88	224.225	0.666	5.88	HBA HYP HYP RA

Null cost = 292.057; fixed cost = 158.138; configuration cost = 17.66.

^a^Cost difference = null cost − total cost.

**Table 2:** The experimental activity and estimated activity of the training set compounds are summarized here.

Compound number	Exp. IC_50_ *μ*M	Estimated IC_50_ *μ*M	Error	Activity magnitude (exp.)	Activity magnitude (est.)	Fit scores
Compound 1	2	0.373	1.627	++	+++	3.981
Compound 2	0.313	0.178	0.135	+++	+++	4.301
Compound 3	0.105	0.127	−0.022	+++	+++	4.449
Compound 4	0.071	0.116	−0.045	++++	+++	4.488
Compound 5	0.34	0.133	0.207	+++	+++	4.427
Compound 6	0.115	0.117	−0.002	+++	+++	4.484
Compound 7	0.19	0.124	0.066	+++	+++	4.457
Compound 8	0.78	0.252	0.528	+++	+++	4.151
Compound 9	0.049	0.202	−0.153	++++	+++	4.247
Compound 10	3.3	2.801	0.499	++	++	3.106
Compound 11	0.36	0.277	0.083	+++	+++	4.11
Compound 12	0.13	0.122	0.008	+++	+++	4.465
Compound 13	0.18	0.177	0.003	+++	+++	4.304
Compound 14	0.14	0.088	0.052	+++	++++	4.609
Compound 15	0.014	0.014	0	++++	++++	5.392
Compound 16	0.9	2.338	−1.438	+++	++	3.185
Compound 17	0.2	0.247	−0.047	+++	+++	4.16
Compound 18	0.07	0.125	−0.055	++++	+++	4.456
Compound 19	0.06	0.058	0.002	++++	++++	4.787
Compound 20	0.08	0.082	−0.002	++++	++++	4.637
Compound 21	0.09	0.126	−0.036	++++	+++	4.451
Compound 22	0.1	0.198	−0.098	++++	+++	4.257
Compound 23	0.5	0.817	−0.317	+++	+++	3.641
Compound 24	0.8	1.744	−0.944	+++	++	3.312
Compound 25	0.039	0.251	−0.212	++++	+++	4.153
Compound 26	0.27	2.13	−1.86	+++	++	3.225
Compound 27	0.043	0.254	−0.211	++++	+++	4.149
Compound 28	3.1	2.181	0.919	++	++	3.215
Compound 29	21	18.8	2.2	+	+	3.213
Compound 30	13	1.52	11.48	+	++	3.372
Compound 31	1.3	1.891	−0.591	++	++	3.277
Compound 32	0.6	0.574	0.026	+++	+++	3.795
Compound 33	10	0.24	9.76	++	+++	4.172
Compound 34	0.032	0.218	−0.186	++++	+++	4.215
Compound 35	3.8	2.305	1.495	++	++	3.191
Compound 36	0.019	0.017	0.002	++++	++++	5.322
Compound 37	0.87	0.232	0.638	+++	+++	4.188
Compound 38	1.48	1.229	0.251	++	++	3.464
Compound 39	3.47	2.075	1.395	++	++	3.237
Compound 40	0.46	2.075	−1.615	+++	++	3.237
Compound 41	0.26	2.115	−1.855	+++	++	3.228
Compound 42	0.56	0.796	−0.236	+++	+++	3.652
Compound 43	5.54	1.82	3.72	++	++	3.293
Compound 44	0.52	0.342	0.178	+++	+++	4.019
Compound 45	1.44	2.101	−0.661	++	++	3.231
Compound 46	0.33	0.773	−0.443	+++	+++	3.665
Compound 47	1.81	0.696	1.114	++	+++	3.711
Compound 48	3.9	2.081	1.819	++	++	3.235

**Table 3:** The experimental and estimated activity of test compounds.

Compound number	Exp. IC_50_ *μ*M	Estimated IC_50_ *μ*M	Error	Activity magnitude (exp.)	Activity magnitude (est.)	Fit scores
Compound 1	3.47	0.174	3.296	++	+++	6.298
Compound 2	10	4.253	5.747	+	++	6.05
Compound 3	0.52	0.009	0.511	+++	++++	5.559
Compound 4	1.81	0.01	1.8	++	++++	5.529
Compound 5	0.1	0.01	0.09	++++	++++	5.518
Compound 6	0.08	0.012	0.068	++++	++++	5.458
Compound 7	0.2	0.015	0.185	+++	++++	5.364
Compound 8	0.9	0.017	0.883	+++	++++	5.309
Compound 9	1.44	0.019	1.421	++	++++	5.272
Compound 10	0.46	0.019	0.441	+++	++++	5.268
Compound 11	1	0.027	0.973	+++	++++	5.116
Compound 12	10	4.033	5.967	+	++	5.097
Compound 13	0.09	0.033	0.057	++++	++++	5.034
Compound 14	5.54	0.36	5.18	++	+++	4.99
Compound 15	0.26	0.046	0.214	+++	++++	4.887
Compound 16	0.33	0.048	0.282	+++	++++	4.865
Compound 17	1.48	0.06	1.42	++	++++	4.771
Compound 18	3	0.202	2.798	++	+++	4.246
Compound 19	0.5	0.239	0.261	+++	+++	4.174
Compound 20	0.05	0.47	−0.42	++++	+++	3.881

## References

[1] Mai A., Massa S., Ragno R. (2003). 3-(4-Aroyl-1-methyl-1*H*-2-pyrrolyl)-*N*-hydroxy-2-alkylamides as a new class of synthetic histone deacetylase inhibitors. 1. Design, synthesis, biological evaluation, and binding mode studies performed through three different docking procedures. *Journal of Medicinal Chemistry*.

[2] Monneret C. (2005). Histone deacetylase inhibitors. *European Journal of Medicinal Chemistry*.

[3] Leipe D. D., Landsman D. (1997). Histone deacetylases, acetoin utilization proteins and acetylpolyamine amidohydrolases are members of an ancient protein superfamily. *Nucleic Acids Research*.

[4] Yang X.-J., Seto E. (2008). The Rpd3/Hda1 family of lysine deacetylases: from bacteria and yeast to mice and men. *Nature Reviews Molecular Cell Biology*.

[5] Bieliauskas A. V., Pflum M. K. H. (2008). Isoform-selective histone deacetylase inhibitors. *Chemical Society Reviews*.

[6] Denu J. M. (2005). The Sir2 family of protein deacetylases. *Current Opinion in Chemical Biology*.

[7] Yang X.-J., Grégoire S. (2005). Class II histone deacetylases: from sequence to function, regulation, and clinical implication. *Molecular and Cellular Biology*.

[8] Gregoretti I. V., Lee Y.-M., Goodson H. V. (2004). Molecular evolution of the histone deacetylase family: functional implications of phylogenetic analysis. *Journal of Molecular Biology*.

[9] Krämer O. H. (2009). HDAC2: a critical factor in health and disease. *Trends in Pharmacological Sciences*.

[10] Kilgore M., Miller C. A., Fass D. M., Hennig K. M., Haggarty S. J., Sweatt J. D., Rumbaugh G. (2010). Inhibitors of class 1 histone deacetylases reverse contextual memory deficits in a mouse model of alzheimer's disease. *Neuropsychopharmacology*.

[11] Wagner J. M., Hackanson B., Lübbert M., Jung M. (2010). Histone deacetylase (HDAC) inhibitors in recent clinical trials for cancer therapy. *Clinical Epigenetics*.

[12] Walkinshaw D. R., Yang X.-J. (2008). Histone deacetylase inhibitors as novel anticancer therapeutics. *Current Oncology*.

[13] Jóna Á., Khaskhely N., Buglio D., Shafer J. A., Derenzini E., Bollard C. M., Medeiros L. J., Illés Á., Ji Y., Younes A. (2011). The histone deacetylase inhibitor entinostat (sndx-275) induces apoptosis in hodgkin lymphoma cells and synergizes with bcl-2 family inhibitors. *Experimental Hematology*.

[14] Younes A., Oki Y., Bociek R. G., Kuruvilla J., Fanale M., Neelapu S., Copeland A., Buglio D., Galal A., Besterman J., Li Z., Drouin M., Patterson T., Ward M. R., Paulus J. K., Ji Y., Medeiros L. J., Martell R. E. (2011). Mocetinostat for relapsed classical Hodgkin's lymphoma: an open-label, single-arm, phase 2 trial. *The Lancet Oncology*.

[15] Prince H. M., Bishton M. J., Harrison S. J. (2009). Clinical studies of histone deacetylase inhibitors. *Clinical Cancer Research*.

[16] Yang S.-Y. (2010). Pharmacophore modeling and applications in drug discovery: challenges and recent advances. *Drug Discovery Today*.

[17] Vadivelan S., Sinha B. N., Rambabu G., Boppana K., Jagarlapudi S. A. R. P. (2008). Pharmacophore modeling and virtual screening studies to design some potential histone deacetylase inhibitors as new leads. *Journal of Molecular Graphics and Modelling*.

[18] Sundarapandian T., Shalini J., Sugunadevi S., Woo L. K. (2010). Docking-enabled pharmacophore model for histone deacetylase 8 inhibitors and its application in anti-cancer drug discovery. *Journal of Molecular Graphics and Modelling*.

[19] Kandakatla N., Ramakrishnan G., Vadivelan S., Jagarlapudi S. (2012). QSAR studies of N-(2-Aminophenyl)-Benzamide derivatives as histone deacetylase2 inhibitors. *International Journal of PharmTech Research*.

[20] Frey R. R., Wada C. K., Garland R. B. (2002). Trifluoromethyl ketones as inhibitors of histone deacetylase. *Bioorganic & Medicinal Chemistry Letters*.

[21] Siliphaivanh P., Harrington P., Witter D. J. (2007). Design of novel histone deacetylase inhibitors. *Bioorganic & Medicinal Chemistry Letters*.

[22] Witter D. J., Harrington P., Wilson K. J., Chenard M., Fleming J. C., Haines B., Kral A. M., Secrist J. P., Miller T. A. (2008). Optimization of biaryl selective HDAC1&2 inhibitors (SHI-1:2). *Bioorganic and Medicinal Chemistry Letters*.

[23] Methot J. L., Chakravarty P. K., Chenard M. (2008). Exploration of the internal cavity of histone deacetylase (HDAC) with selective HDAC1/HDAC2 inhibitors (SHI-1:2). *Bioorganic & Medicinal Chemistry Letters*.

[24] Wilson K. J., Witter D. J., Grimm J. B., Siliphaivanh P., Otte K. M., Kral A. M., Fleming J. C., Harsch A., Hamill J. E., Cruz J. C., Chenard M., Szewczak A. A., Middleton R. E., Hughes B. L., Dahlberg W. K., Secrist J. P., Miller T. A. (2008). Phenylglycine and phenylalanine derivatives as potent and selective HDAC1 inhibitors (SHI-1). *Bioorganic and Medicinal Chemistry Letters*.

[25] Tessier P., Smil D. V., Wahhab A. (2009). Diphenylmethylene hydroxamic acids as selective class IIa histone deacetylase inhibitors. *Bioorganic & Medicinal Chemistry Letters*.

[26] Raeppel S., Zhou N., Gaudette F. (2009). SAR and biological evaluation of analogues of a small molecule histone deacetylase inhibitor *N*-(2-aminophenyl)-4-((4-(pyridin-3-yl)pyrimidin-2-ylamino)methyl)benzamide (MGCD0103). *Bioorganic & Medicinal Chemistry Letters*.

[27] Heidebrecht R. W., Chenard M., Close J., Dahlberg W. K., Fleming J., Grimm J. B., Hamill J. E., Harsch A., Haines B. B., Hughes B., Kral A. M., Middleton R. E., Mushti C., Ozerova N., Szewczak A. A., Wang H., Wilson K., Witter D. J., Secrist J. P., Miller T. A. (2009). Exploring the pharmacokinetic properties of phosphorus-containing selective HDAC 1 and 2 inhibitors (SHI-1:2). *Bioorganic and Medicinal Chemistry Letters*.

[28] Butler K. V., Kozikowski A. P. (2008). Chemical origins of isoform selectivity in histone deacetylase inhibitors. *Current Pharmaceutical Design*.

[29] Jones P., Steinkühler C. (2008). From natural products to small molecule ketone histone deacetylase inhibitors: development of new class specific agents. *Current Pharmaceutical Design*.

[30] Zuomei L., Murakami K. Combination therapy.

[31] Bressi J. C., Jennings A. J., Skene R., Wu Y., Melkus R., Jong R. D., O'Connell S., Grimshaw C. E., Navre M., Gangloff A. R. (2010). Exploration of the HDAC2 foot pocket: synthesis and SAR of substituted *N*-(2-aminophenyl)benzamides. *Bioorganic and Medicinal Chemistry Letters*.

[32] Joseph J. B., Sriram B. Uses of selective inhibitors of hdac8 for treatment of t-cell proliferative disorders.

[33] Smil D. V., Manku S., Chantigny Y. A. (2009). Novel HDAC6 isoform selective chiral small molecule histone deacetylase inhibitors. *Bioorganic & Medicinal Chemistry Letters*.

[34] Giannini G., Marzi M., Marzo M. D., Battistuzzi G., Pezzi R., Brunetti T., Cabri W., Vesci L., Pisano C. (2009). Exploring bis-(indolyl)methane moiety as an alternative and innovative CAP group in the design of histone deacetylase (HDAC) inhibitors. *Bioorganic and Medicinal Chemistry Letters*.

[35] Li H., Sutter J., Hoffmann R., Güner O. F. HypoGen: an automated system for generating predictive 3D pharmacophore models.

[36] Langer T., Wolber G. (2004). Pharmacophore definition and 3D searches. *Drug Discovery Today: Technologies*.

[37] http://dtp.nci.nih.gov/.

[38] Maybride Database http://www.maybridge.com/.

[39] Venkatachalam C. M., Jiang X., Oldfield T., Waldman M. (2003). LigandFit: a novel method for the shape-directed rapid docking of ligands to protein active sites. *Journal of Molecular Graphics and Modelling*.

[40] Noureen N., Kalsoom S., Rashid H. (2010). Ligand based pharmacophore modelling of anticancer histone deacetylase inhibitors. *African Journal of Biotechnology*.

