# Supplementary material for: Ligand Based Pharmacophore Modeling and Virtual Screening Studies to Design Novel HDAC2 Inhibitors
Source: Adv Bioinformatics. 2014 Nov 26;2014:812148. doi: 10.1155/2014/812148 (PMC4265523; doi:10.1155/2014/812148)
Supplement: Supplementary file 1 — Supplementary figures 1 and 2 provides the chemical structures of training and test set compounds, Supplementary Table 1 provide the randomization test results, Supplementary Table 2-4 provides the docking score, H-bond interactions and length, amino acid residues of Entinostat and 20 lead compounds on HDAC2 receptor and Supplementary Table 5-6 provides the docking score, H-bond interactions and length, amino acid residues of Entinostat and 8 lead compounds on HDAC and HDAC8 receptor. [file 812148.f1.docx]

Supplementary figures 1 and 2 provides the chemical structures of training and test set compounds, Supplementary Table 1 provide the randomization test results, Supplementary Table 2-4 provides the docking score, H-bond interactions and length, amino acid residues of Entinostat and 20 lead compounds on HDAC2 receptor and Supplementary Table 5-6 provides the docking score, H-bond interactions and length, amino acid residues of Entinostat and 8 lead compounds on HDAC and HDAC8 receptor

Compound1 (2µM) Compound2 (0.313µM) Compound3 (0.105 µM)

Compound4 (0.071µM) Compound5 (0.34µM) Compound6 (0.115µM )

Compound7 (0.19µM) Compound8 (0.78µM) Compound9 (0.049µM)

Compound10(3.3µM) Compound11(0.36µM) Compound12(0.13µM) Compound13(0.18µM)

Compound14 (0.14µM) Compound15 (0.014µM) Compound16 (0.9µM)

Compound17(0.2µM) Compound18(0.07µM) Compound19(0.06µM)

Compound20(0.08µM) Compound21(0.09µM) Compound22(0.1µM)

Compound23(0.5µM) Compound24(0.8µM) Compound25(0.039µM) Compound26(0.27µM)

Compound27(0.043µM) Compound28(3.1µM) Compound29(21µM) Compound30(13µM) Compound31(1.3µM) Compound32(0.6µM) Compound33(10µM) Compound34(0.032µM) Compound35(3.8µM) Compound36(0.019µM) Compound37(0.87µM) Compound38(1.48µM)

Compound39(3.47µM) Compound40(0.46µM) Compound41(0.26µM) Compound42 (0.56µM)

Compound43(5.54µM) Compound44(0.52µM) Compound45(1.44µM) Compound46 (0.33µM)

Compound47 (1.81µM) Compound48 (3.9µM)

Fig.1. Training set of compounds with inhibitory (IC_50_ µM) activity

Compound1 (3.47µM) Compound2 (10µM) Compound3 (0.52µM) Compound4 (1.81µM)

Compound5 (0.1µM) Compound6 (0.08µM) Compound7 (0.2µM) Compound8 (0.9µM)

Compound9 (1.44µM) Compound10 (0.46µM) Compound11 (1.0µM)

Compound12 (10µM) Compound13 (0.09µM) Compound14 (5.54µM)

Compound15 (0.26µM) Compound16 (0.33µM) Compound17 (1.48µM)

Compound18 (3µM) Compound19 (0.5µM) Compound20 (0.05µM)

Fig.2. Test set of compounds with inhibitory activity (IC_50_)

Table 1: Results from Randomization test

| Validation No | Total cost | Correlation | Cost difference |
| --- | --- | --- | --- |
| Hypo1 | 223.598 | 0.759 | 68.459 |
| Random1 | 283.616 | 0.42 | 8.441 |
| Random2 | 249.75 | 0.638 | 42.307 |
| Random3 | 269.434 | 0.493 | 22.623 |
| Random4 | 292.057 | 1.254 | 0 |
| Random5 | 279.363 | 0.425 | 12.694 |
| Random6 | 261.573 | 0.549 | 30.484 |
| Random7 | 283.221 | 0.331 | 8.836 |
| Random8 | 271.033 | 0.492 | 21.024 |
| Random9 | 273.469 | 0.458 | 18.588 |
| Random10 | 259.068 | 0.589 | 32.989 |
| Random11 | 292.057 | 1.173 | 0 |
| Random12 | 270.203 | 0.472 | 21.854 |
| Random13 | 272.849 | 0.476 | 19.208 |
| Random14 | 282.555 | 0.419 | 9.502 |
| Random15 | 262.085 | 0.557 | 29.972 |
| Random16 | 258.684 | 0.573 | 33.373 |
| Random17 | 292.057 | 1.341 | 0 |
| Random18 | 279.913 | 0.454 | 12.144 |
| Random19 | 276.828 | 0.432 | 15.229 |

Table 2: The docking score of Entinostat with HDAC2

| HDAC2 (3MAX) | Chain-A | | Chain-B | | Chain-C | |
| --- | --- | --- | --- | --- | --- | --- |
|  | Docking score (kcal/mol) | H-bond interaction | Docking score (kcal/mol) | H-bond interaction | Docking score (kcal/mol) | H-bond interaction |
| MS-275 (Entinostat) | 42.653 | Arg39, Cys156, Gly305, His183 | 39.07 | Tyr308, tyr29 | 36.9 | Tyr308, tyr29 |

Table 3: Virtually identified novel HDAC2 leads with docking score

| Compound Name | LigScore1 | LigScore2 | -PLP1 | -PLP2 | -PMF | Dock Score | Lig-Interaction energy | Estimated activity |
| --- | --- | --- | --- | --- | --- | --- | --- | --- |
| NCI database | | | | | | | | |
| NSC86319 | 5.84 | 3.47 | 92.95 | 77.58 | 123.96 | 126.904 | -3.101 | 0.172 |
| NSC108392 | 5.7 | 4.94 | 87.95 | 89.23 | 126.66 | 121.924 | -2.96 | 0.267 |
| NSC127064 | 5.97 | 4.66 | 134.4 | 125.11 | 150.33 | 116.487 | 0.664 | 0.475 |
| NSC86310 | 6.1 | 3.8 | 105.08 | 91.22 | 115.72 | 109.1 | -2.606 | 0.661 |
| NSC110782 | 3.93 | 3.69 | 95.01 | 89.46 | 134.03 | 106.256 | -4.101 | 0.372 |
| NSC748337 | 5.58 | 4.72 | 112.76 | 122.42 | 163.25 | 105.669 | -4.03 | 0.416 |
| NSC748338 | 4.82 | 4.08 | 114.54 | 126.51 | 160.93 | 102.156 | -3.641 | 0.519 |
| NSC210292 | 3.82 | 4.56 | 111.38 | 110.15 | 145.3 | 82.415 | 2.805 | 0.552 |
| NSC169121 | 3.61 | 4.99 | 90.09 | 94.21 | 148.61 | 80.3 | 5.705 | 0.61 |
| NSC747475 | 4.12 | 4.6 | 121.91 | 122.02 | 151.94 | 80.504 | -0.011 | 0.747 |
| Maybridge database | | | | | | | | |
| MFCD01935795 | 4.96 | 4.95 | 81.9 | 89.99 | 110.57 | 98.831 | 0.258 | 0.124 |
| MFCD00205730 | 5.07 | 5.19 | 96.57 | 104.51 | 97.1 | 97.191 | -0.852 | 0.206 |
| MFCD00830779 | 3.12 | 4.45 | 92.76 | 79.47 | 92.26 | 96.892 | -1.697 | 0.323 |
| MFCD00276929 | 5.01 | 5.09 | 83.11 | 92.14 | 108.93 | 95.218 | 4.622 | 0.462 |
| MFCD00277189 | 5.14 | 5.66 | 122.24 | 120.48 | 162.91 | 92.377 | 5.171 | 0.675 |
| MFCD00661790 | 5.81 | 5.34 | 104.1 | 105.06 | 156.1 | 81.47 | -0.79 | 0.614 |
| MFCD00174795 | 5.19 | 4.96 | 85.82 | 94.82 | 111.49 | 80.032 | -0.495 | 0.572 |
| MFCD04123099 | 5.07 | 5.1 | 85.86 | 90.55 | 123.57 | 77.73 | 8.624 | 0.858 |
| MFCD00124221 | 3.83 | 4.65 | 97.96 | 94.36 | 125.24 | 65.862 | -3.219 | 0.687 |
| MFCD00116804 | 5.38 | 5.51 | 103.48 | 96.6 | 129.05 | 61.148 | 14.943 | 0.689 |

Table 4: Identified lead compounds with H-bond interaction

| Compound Name | H-bond interaction | H-bond monitoring | H-bond distance |
| --- | --- | --- | --- |
| NCI database | | | |
| NSC86319 | Arg39 (2) | A:ARG39:HE - 86319:N28 A:ARG39:HH21 - 86319:N28 | 1.94, 2.01 |
| NSC108392 | Arg39 (3), His145, Asp181 (2) | A:ARG39:HE - 108392:N23 A:ARG39:HH21 - 108392:O22 A:ARG39:HH21 - 108392:N23 108392:H37 - A:HIS145:NE2 108392:H37 - A:ASP181:OD1 108392:H38 - A:ASP181:OD2 | 2.41, 2.34, 1.76, 2.09,2.05, 2.39 |
| NSC127064 | Arg39, Cys156, Gly305, His 145 (2), Asp181 (2), Trp140, Gly142 | A:ARG39:HH22 - 127064:O27 A:CYS156:HG - 127064:O24 A:GLY305:HN - 127064:O27 127064:H43 - A:HIS145:NE2 127064:H43 - A:ASP181:OD2 127064:H45 - A:HIS145:NE2 127064:H45 - A:ASP181:OD1 127064:H48 - A:TRP140:O 127064:H48 - A:GLY142:O | 2.21, 2.13, 2.13, 2.46, 2.12, 2.39, 2.07, 2.33, 2.49 |
| NSC86310 | His145, Asp181 | 86310:H40 - A:HIS145:NE2 86310:H40 - A:ASP181:OD2 | 2.48, 2.16 |
| NSC110782 | His145, Asp181 (3), Gly154, Ala141 | 110782:H40 - A:HIS145:NE2 110782:H40 - A:ASP181:OD1 110782:H41 - A:ASP181:OD1 110782:H41 - A:ASP181:OD2 110782:H42 - A:GLY154:O 110782:H43 - A:ALA141:O | 2.16, 1.95, 2.38, 2.17, 1.78, 1.83 |
| NSC748337 | Asp181 (2), His145, Ala141, His183 | 748337:H45 - A:ASP181:OD2 748337:H46 - A:HIS145:NE2 748337:H46 - A:ASP181:OD1 748337:H48 - A:ALA141:O A:HIS183:HD1 - 748337:N1 | 1.95, 2.10, 2.46, 1.71, 1.99 |
| NSC748338 | His183, Asp181 (2), Ala141 | A:HIS183:HD1 - 748338:N1 748338:H45 - A:ASP181:OD2 748338:H46 - A:ASP181:OD1 748338:H49 - A:ALA141:O | 2.00, 1.95, 2.04, 1.90 |
| NSC210292 | Asp104, Gly154 | 210292:H44 - A:ASP104:OD2 210292:H46 - A:GLY154:O | 1.86, 2.31 |
| NSC169121 | Cys156, Gly143 | A:CYS156:HG - Molecule-1:O14 Molecule-1:H48 - A:GLY143:O | 1.94, 2.48 |
| NSC747475 | Arg39 (2), Gly154 | A:ARG39:HH21 - 747475:O6 A:ARG39:HH22 - 747475:O6 747475:H53 - A:GLY154:O | 2.15, 2.29, 2.10 |
| Maybridge database | | | |
| MFCD01935795 | CYS156, PHE155, HIS146 | A:CYS156:HG - Compound_Number_45008:N1 Compound_Number_45008:H36 - A:PHE155:O Compound_Number_45008:H37 - A:HIS146:NE2 | 2.24, 2.12, 1.98 |
| MFCD00205730 | ARG39, HIS146 | A:ARG39:HH21 - Compound_Number_56643:F15 Compound_Number_56643:H36 - A:HIS146:NE2 | 2.47, 1.82 |
| MFCD00830779 | ARG39, HIS183, ASP181 (2), ASP269, ASP181, HIS146 | A:ARG39:HH21 - Compound_Number_28196:N10 A:HIS183:HD1 - Compound_Number_28196:O18 Compound_Number_28196:H29 - A:ASP181:OD2 Compound_Number_28196:H30 - A:ASP269:OD2 Compound_Number_28196:H31 - A:ASP181:OD1 Compound_Number_28196:H32 - A:HIS146:NE2 | 2.29, 2.49, 1.86, 1.68, 1.75, 1.91 |
| MFCD00276929 | CYS156, PHE155, HIS146 | A:CYS156:HG - Compound_Number_45007:N1 Compound_Number_45007:H33 - A:PHE155:O Compound_Number_45007:H34 - A:HIS146:NE2 | 2.23, 2.08, 1.92 |
| MFCD00277189 | HIS146 (2) | Compound_Number_38762:H47 - A:HIS146:NE2 Compound_Number_38762:H50 - A:HIS146:NE2 | 1.92, 2.05 |
| MFCD00661790 | CYS156, HIS183, HIS146, ALA141 | A:CYS156:HG - Compound_Number_567:O17 Compound_Number_567:H39 - A:HIS183:NE2 Compound_Number_567:H41 - A:HIS146:NE2 Compound_Number_567:H43 - A:ALA141:O | 2.26, 2.22, 2.08, 1.79 |
| MFCD00174795 | CYS156, PHE155, HIS146 | A:CYS156:HG - Compound_Number_45320:N1 Compound_Number_45320:H39 - A:PHE155:O Compound_Number_45320:H41 - A:HIS146:NE2 | 2.25, 2.20, 1.92 |
| MFCD04123099 | CYS156, PHE155, HIS146 | A:CYS156:HG - Compound_Number_45111:N1 Compound_Number_45111:H36 - A:PHE155:O Compound_Number_45111:H38 - A:HIS146:NE2 | 2.23, 2.02, 1.92 |
| MFCD00124221 | CYS156, GLY305, HIS183 | A:CYS156:HG - Compound_Number_38764:O4 Compound_Number_38764:H41 - A:GLY305:O Compound_Number_38764:H42 - A:HIS183:NE2 | 2.03, 2.43, 1.97 |
| MFCD00116804 | ARG39 (2), HIS146 | A:ARG39:HH21 - Compound_Number_3618:O18 A:ARG39:HH22 - Compound_Number_3618:O18 Compound_Number_3618:H40 - A:HIS146:NE2 | 2.14, 2.27, 1.99 |

Table 5: The docking score of Entinostat with HDAC (1ZZ1) and HDAC8 (1T64)

| HDAC (1ZZ1) | ChainA | | ChainB | | ChainC | | ChainD | |
| --- | --- | --- | --- | --- | --- | --- | --- | --- |
|  | Docking score (kcal/mol) | H-bond interaction | Docking score (kcal/mol) | H-bond interaction | Docking score (kcal/mol) | H-bond interaction | Docking score (kcal/mol) | H-bond interaction |
| Entinostat | 35.302 | Asp15, Lys43, Gly104, Asn138, Asn105 | 21.06 | Lys36, Lys33 | 33.486 | Ile100, Thr92 | 33.205 | Thr18 (2), Met102 |
| HDAC8 (1T64) | ChainA | | ChainB | |  |  |  |  |
|  | Docking score (kcal/mol) | H-bond interaction | Docking score (kcal/mol) | H-bond interaction |  |  |  |  |
| Entinostat | 24.99 | Ser26, Glu335, Ser39 | 23.672 | Gly17, Asp41(2) |  |  |  |  |

Table 6: Docking score and H-bond interactions of Entinostat and four lead compounds

| Protein | Compound | Docking score | H-bond interaction | H-bond monitoring | H-bond distance |
| --- | --- | --- | --- | --- | --- |
| HDAC (1ZZ1) | MS-275 | 35.302 | ASP15, LYS43, GLY104, ASN138, ASN105 | A:ASP15:HN - Compound3:O7 A:LYS43:HZ3 - Compound3:N11 A:GLY104:HN - Compound3:O7 A:ASN138:HD22 - Compound3:N11 Compound3:H46 - A:ASN105:O | 2.43, 2.00, 2.37, 2.11, 1.81 |
|  | NSC108392 | 60.935 | ALA23, LEU21, THR92 (2) | A:ALA23:HN - Compound5:N1 Compound5:H37 - A:LEU21:O Compound5:H40 - A:THR92:O Compound5:H41 - A:THR92:OG1 | 2.39, 1.89, 1.79, 1.88 |
|  | NSC127064 | 78.519 | LEU21 (2), HIS36, PHE152, MET102 | Compound15:H44 - A:LEU21:O Compound15:H46 - A:LEU21:O Compound15:H46 - A:HIS36:NE2 Compound15:H48 - A:PHE152:O Compound15:H49 - A:MET102:O | 2.14, 2.03, 2.33, 2.08, 1.92 |
|  | NSC110782 | 70.278 | ASN105, LEU21, HIS36, THR92 | A:ASN105:HN - Compound20:O22 Compound20:H40 - A:LEU21:O Compound20:H40 - A:HIS36:NE2 Compound20:H44 - A:THR92:O | 2.07, 2.00, 2.27, 1.95 |
|  | MFCD01935795 | 47.07 | ASP15, GLY104 (2), PHE152 | A:ASP15:HN - Maybridge_leads.cdx:N12 A:GLY104:HN - Maybridge_leads.cdx:N12 A:GLY104:HN - Maybridge_leads.cdx:O20 Maybridge_leads.cdx:H37 - A:PHE152:O | 2.10, 2.30, 2.05 |
|  | MFCD00830779 | 54.69 | LEU21 (2), HIS36, ILE100, THR16 | Maybridge_leads.cdx:H29 - A:LEU21:O Maybridge_leads.cdx:H30 - A:LEU21:O Maybridge_leads.cdx:H30 - A:HIS36:NE2 Maybridge_leads.cdx:H31 - A:ILE100:O Maybridge_leads.cdx:H32 - A:THR16:O | 2.03, 2.40, 1.67, 2.45, 1.66, 1.69 |
|  | MFCD00661790 | 45.78 | ALA38, ILE100 (2), LEU21, HIS36 | Maybridge_leads.cdx:H39 - A:ALA38:O Maybridge_leads.cdx:H41 - A:ILE100:O Maybridge_leads.cdx:H42 - A:LEU21:O Maybridge_leads.cdx:H42 - A:HIS36:NE2 Maybridge_leads.cdx:H43 - A:ILE100:O | 2.41, 2.19, 1.95, 2.02, 2.11 |
|  | MFCD00124221 | 33.15 | ASP15 (3) | Maybridge_leads.cdx:H42 - A:ASP15:OD2 Maybridge_leads.cdx:H43 - A:ASP15:OD1 Maybridge_leads.cdx:H43 - A:ASP15:OD2 | 1.79, 2.19, 2.19 |
| HDAC8 (1T64) | MS-275 | 24.99 | SER26, GLU335, SER39 | A:SER26:HG - Compound3:O8 Compound3:H46 - A:GLU335:OE2 Compound3:H47 - A:SER39:OG | 1.90, 1.98, 1.94 |
|  | NSC108392 | 66.209 | TYR111 (2), TYR306 | Compound5:H37 - A:TYR111:OH Compound5:H38 - A:TYR111:OH Compound5:H40 - A:TYR306:O | 1.92, 1.93, 1.82 |
|  | NSC127064 | 55.377 | ARG37 (3), PRO35, CYS28 | A:ARG37:HN - Compound15:O24 A:ARG37:HE - Compound15:O27 Compound15:H47 - A:PRO35:O Compound15:H48 - A:CYS28:O | 2.30, 2.43, 1.64, 1.72, 1.78 |
|  | NSC110782 | 63.86 | PHE152, TYR111, LYS33 | Compound19:H40 - A:PHE152:O Compound19:H41 - A:TYR111:OH Compound19:H44 - A:LYS33:O | 2.05, 1.78, 1.87 |
|  | NSC748337 | 55.228 | TYR111 | Compound34:H48 - A:TYR111:OH | 1.71 |
|  | MFCD01935795 | 54.29 | TYR111 (3), CYS28 | A:TYR111:HH - Maybridge_leads.cdx:N9 Maybridge_leads.cdx:H36 - A:TYR111:OH Maybridge_leads.cdx:H37 - A:TYR111:OH Maybridge_leads.cdx:H38 - A:CYS28:O | 2.34, 2.46, 1.92, 1.95 |
|  | MFCD00830779 | 54.56 | TYR111 (2), CYS28, PRO35 | A:TYR111:HH - Maybridge_leads.cdx:O10 Maybridge_leads.cdx:H29 - A:CYS28:O Maybridge_leads.cdx:H30 - A:PRO35:O Maybridge_leads.cdx:H32 - A:TYR111:OH | 2.24, 1.82, 1.65, 2.16 |
|  | MFCD00661790 | 41.84 | CYS28 | Maybridge_leads.cdx:H39 - A:CYS28:O | 1.78 |
|  | MFCD00124221 | 29.54 | LYS36 | A:LYS36:HZ1 - Maybridge_leads.cdx:O10 | 1.42 |
